# Self-Healing Bio-Concrete Using *Bacillus subtilis* Encapsulated in Iron Oxide Nanoparticles

**DOI:** 10.3390/ma15217731

**Published:** 2022-11-03

**Authors:** Faisal Mahmood, Sardar Kashif Ur Rehman, Mohammed Jameel, Nadia Riaz, Muhammad Faisal Javed, Abdelatif Salmi, Youssef Ahmed Awad

**Affiliations:** 1 Department of Civil Engineering, COMSATS University Islamabad, Abbottabad Campus, Abbottabad 22060, Pakistan; 2 Department of Civil Engineering, King Khalid University, Abha 61421, Saudi Arabia; 3 Department of Environmental Sciences, COMSATS University Islamabad, Abbottabad Campus, Abbottabad 22060, Pakistan; 4 Department of Civil Engineering, College of Engineering in Alkharj, Prince Sattam bin Abdulaziz University, Alkharj 16273, Saudi Arabia; 5 Structural Engineering Department, Faculty of Engineering and Technology, Future University in Egypt, New Cairo 11835, Egypt

**Keywords:** iron oxide nanoparticles, *Bacillus subtilis*, bio-concrete, crack healing, microbially induced calcium carbonate

## Abstract

For the creation of healable cement concrete matrix, microbial self-healing solutions are significantly more creative and potentially successful. The current study investigates whether gram-positive “*Bacillus subtilis*” (*B. subtilis*) microorganisms can effectively repair structural and non-structural cracks caused at the nano- and microscale. By creating an effective immobilization strategy in a coherent manner, the primary challenge regarding the viability of such microbes in a concrete mixture atmosphere has been successfully fulfilled. The iron oxide nanoparticles were synthesized. The examined immobilizing medium was the iron oxide nanoparticles, confirmed using different techniques (XRD, SEM, EDX, TGA, and FTIR). By measuring the average compressive strength of the samples (ASTM C109) and evaluating healing, the impact of triggered *B. subtilis* bacteria immobilized on iron oxide nanoparticles was examined. The compressive strength recovery of cracked samples following a therapeutic interval of 28 days served as a mechanical indicator of the healing process. In order to accurately correlate the recovery performance as a measure of crack healing duration, the pre-cracking load was set at 80% of the ultimate compressive stress, or “f c,” and the period of crack healing was maintained at 28 days. According to the findings, *B. subtilis* bacteria greatly enhanced the compressive strength and speed up the healing process in cracked cement concrete mixture. The iron oxide nanoparticles were proven to be the best immobilizer for keeping *B. subtilis* germs alive until the formation of fractures. The bacterial activity-driven calcite deposition in the generated nano-/micro-cracks was supported by micrographic and chemical investigations (XRD, FTIR, SEM, and EDX).

## 1. Introduction

**Figure 1:**
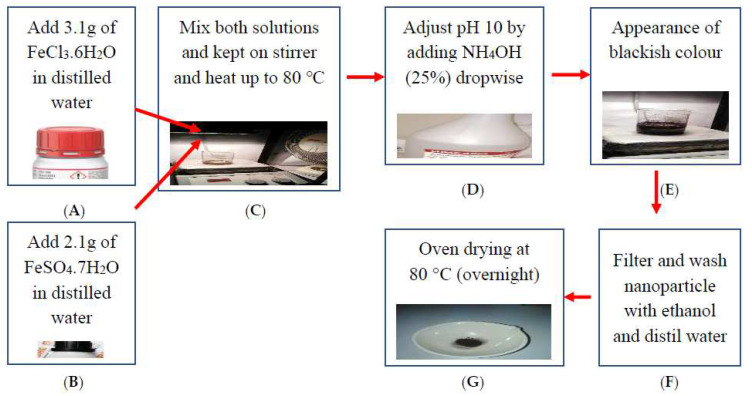
Precipitation method for synthesis of IONPs. (**A**) Adding FeCl_3_.6H_2_O in distilled water; (**B**) Adding FeSO_4_.7H_2_O in distilled water; (**C**) Mixing and stirring both solutions; (**D**) pH adjustment; (**E**) Blackish colour appearance; (**F**) Filtering and washing; and (**G**) Oven drying.

Concrete has become one of the world’s most widely utilized substances due to its unique properties, which include good compressive strength, proximity, adaptability, economically, suitability with reinforced steel bar, flame retardation, superior caloric weight, and its ability to be cast in specific forms and dimensions [1,2]. Concrete, on the other hand, is prone to cracking, and these fissures, which are caused by mechanical and environmental forces, considerably degrade the structure’s performance, steel corrosion [3], serviceability, and longevity [4]. Despite the fact that passive crack treatments (such as chemical and polymer sealants) are available in the market [5], they are typically time-consuming and non-sustainable [6]. The spalling of concrete and rusting of reinforcing bars accelerate the deterioration of concrete structures as a result of ongoing crack growth [7,8,9]. High replacement costs and environmental problems are linked to these phenomena [10,11]. As a result, a realistic solution to the concrete cracking problem that does not compromise mechanical qualities is required. Microorganisms’ intrinsic ability to manufacture calcium carbonate precipitation has inspired biotechnological approaches for the production of a new creation of auto-healing concrete [12]. When a split forms, bacteria within it are triggered, causing calcium carbonate minerals to form, which close the split. In comparison to traditional chemical self-healing concrete, the microbially induced calcium carbonate (MICP)-based bio self-healing technique provides a long-term and ecologically benign solution to cracking [13,14,15]. MICP production occurs in the presence of CO_2_, which is basically produced from the precursor used in bio-concrete. Different types of precursors are used by scientists to obtain CaCO_3_. The most common precursors include amino acid salt [16,17] and urea [18,19]. The autotrophic microbes turn CO_2_ to CaCO_3_, which is basically produced when water comes into contact with the urea or salt of the amino acid given in Equation (1). When there is Ca^+2^ in the surrounding atmosphere, the negatively charged bacterial cell membrane will pull positive charges from the surroundings after the Ca^+2^ is in the process of demineralization. Calcium ions (Ca^+2^) will ultimately be collected on the surface of the cell, as shown in Figure 1. After that, the Ca^+2^ ions integrate with the CO_3_^−2^ ions, resulting in CaCO_3_ precipitation, as shown in Equation (2).

CO(NH_2_)_2_ + H_2_O → CO_2_ + 2NH_3_ (1)

Ca^+2^ + CO_3_^2−^ → CaCO_3_ (2)

Calcium carbonate is made outside of the cell membranes of bacteria via autotrophic and heterotrophic metabolic processes. The autotrophic microbes convert the CO_2_ to CaCO_3_ by non-methylotrophic methanogenesis, aerobic photosynthesis, and anaerobic photosynthesis using different bacteria in a variety of environments. MICP has been discovered in the formation of calcium carbonate in a variety of geological environments, including soils, limestone caves, oceans, and soda lakes [20]. Biotic mechanisms can produce calcium carbonate minerals in two ways: autotrophic and heterotrophic processes [21]. Using MICP-based self-healing technology to address engineering, geotechnical, and environmental concerns has evolved as a realistic, sustainable, and repeatable solution over the last decade [16,17,19,22,23]. The use of MICP-based techniques to adapt and improve construction substances (quality and production) such as concrete and mortar has caught the interest of researchers [16,17,19]. 

The number and quality of induced minerals have a significant impact on the efficacy of MICP processes [15,24]. For example, the maximum deposition of calcium carbonate leads to more filling of fractures, which improves the durability performance of bio-healing concrete. Calcium carbonate comes in a variety of polymorphs, each with its own set of physical and mechanical properties that have an influence on the characteristics of deposition [25,26]. The purpose of this study was to determine the consequences of IONP’s encapsulated bacterial spores on the crack healing and strength recovery and to analyze the water absorption of bacterial concrete, to improve the performance of bio-concrete as well as the existing hurdles of implementing this breakthrough technology in situ. The synthesis and characterization of IONPs and the characterization of MICP products (CaCO_3_) were also discussed in the paper.

## 2. Materials and Methods

### 2.1. Materials

The bacterial concrete used in this research consisted of the commercial calcium lactate, purchased from Sigma-Aldrich, Schnelldorf, Germany. The chemicals included FeCl_3_.6H_2_O, FeCl_2_.4H_2_O, sodium hydroxide, MnSO_4_.H_2_O (Sigma Aldrich > 99% pureness), ethanol (Merck > 99–100% purity). Reagents were used during the synthesis, characterization, and reaction study, with no further purification.

For the casting of the bacterial concrete, the bacteria *Bacillus subtilis* was isolated from soil using a streak plate technique. Ordinary Portland cement (Grade 53) was purchased from Fauji Cement Company (Islamabad, Pakistan) (Pakistan Standard PS-232-2008—Grade 53, which means that the compressive strength of the cement after 28 days is 53 N/mm^2^). The iron oxide nanoparticles were synthesized in the laboratory using a chemical precipitation method. Margalla coarse aggregates were used in this research. The calcium lactate used was a high purity product purchased from Sigma-Aldrich, Germany. The urea, calcium chloride anhydrous, and ammonium hydroxide were purchased from Daejung, Siheung si, Korea. The yeast extract, nutrient broth, and nutrient agar were purchased from Oxoid (Hampshire, UK). Deionized distilled water was produced in the laboratory using a B114 deionizer.

### 2.2. Synthesis and Characterization of Iron Oxide Nanoparticles (IONPs)

The chemical precipitation method was used to synthesize the IONPs. A schematic diagram is shown in Figure 1. The addition of 3.1 g of FeCl_3_.6H_2_O in distilled water was designated as solution A. In addition, a second solution B was generated by the addition of 2.1 g of FeSO_4_.7H_2_O in distilled water. Under intense stirring, solution B was added dropwise to solution A and heated up to 80 °C. Soon after, the appearance of the color black was a sign of the IONPs. The nanoparticles were filtered and washed with ethanol and distilled water thrice. The precipitates were then dried in an oven (UN 30, Memmert Germany, Schwabach, Germany) overnight at 80 °C. 

The characterization of the IONPs was crucial to better understand the physio-chemical properties. The performance of these nanoparticles can be best explained based on these properties as a nanopellet for the encapsulation of bacteria when put into the concrete mix during casting. The information derived from different characterization techniques can be helpful for understanding the relationship between the physicochemical characteristics and their efficiency as carriers of bacteria. The thermal stability of IONPs was also checked. As IONPs were added to the concrete mix, the temperature within the concrete raised to 80–90 °C due to hydration reactions [27]. Therefore, it was necessary to check the thermal stability of the IONPs. Moreover, IONPs are carriers of bacteria and they act as a shield for bacteria under high temperatures. Therefore, the IONPs were characterized for thermal stability using thermal gravimetric analysis (TGA; STA 8000, Boston, MA, USA). For this purpose, 20 mg of the synthesized dried IONP powder was used. The IONPs were subjected to an incremental heating rate of 10 °C min^−1^ until reaching 800 °C from the ambient temperature. The results were reported as the percent weight loss against the relative temperature. Functional groups in the synthesized nanomaterial were identified and compared using an Alpha Bruker, FTIR Spectrophotometer (Karlsruhe, Germany). The nanomaterials were pressed into pellets and scanned at 4000 to 400 cm^−1^. The procedure for obtaining the FTIR spectra was as follows. The synthesized sample of the IONPs was heated for 24 h at 80 °C in an oven in a controlled environment. The obtained IONPs were placed in a sample container. Spectrum analysis software was used to acquire the infrared spectrum. For analysis of the white healing precipitate, samples were extracted from the broken pieces of bio-concrete. The white precipitated samples of 30 mg were dried first in an oven at 50 °C and then ground into a fine powder < 5 μm in size. 

The crystalline sizes of the IONPs were measured using X-ray diffraction (XRD- Bruker, Billerica, MA, USA). The pattern of XRD was scanned at a scan rate of 2° per min from 20–80° using a Cu Kα radiation source. The IONP samples were collected in fine powder form. For the characterization of the healing material, the test samples were collected from the outer surface of the bio-concrete. Moreover, the healing material from remainant pieces of healed bio-concrete samples after the compression test were obtained and then ground into a fine powder < 5 μm. The crystalline size was estimated using the Scherrer equation [28].

(3) $$D=\frac{K\lambda }{\beta \mathrm{Cos}\theta }$$

where K is the Scherrer constant, λ represents the radiation wavelength, θ exhibits the diffraction angle of the rays, and β is the full width at half maximum (FWHM).

Surface morphology was conducted with scanning electron spectroscopy using a JEOL, JSM-6510LA, Tokyo, Japan. Scanning electron microscopy [29] was used to determine the morphology and elemental analysis of the IONP sample. SEM analysis was carried out on the JEOL, JSM-6510LA microscope. The IONP samples were coated with a conductive material and placed on the studs for analysis in the sample chamber of the instrument. Images were recorded at different magnifications and distances.

### 2.3. Microorganisms’ Isolation, Identification, and Growth Medium

**Figure 2:**
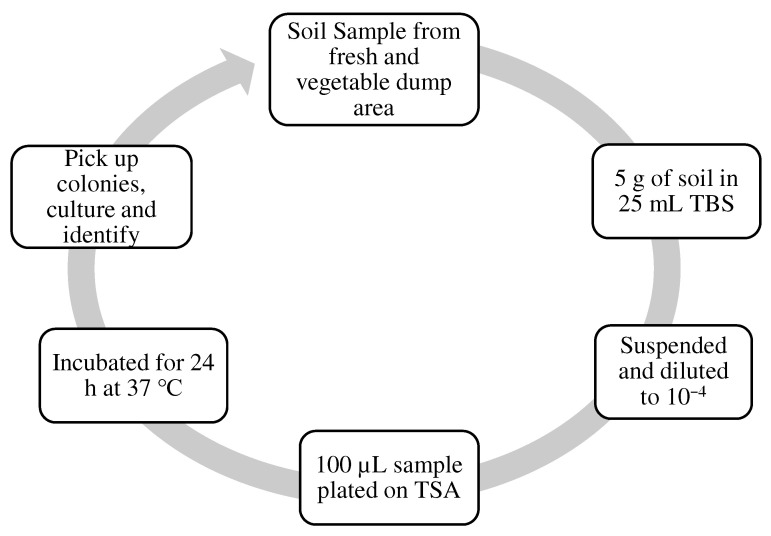
Isolation technique applied for bacteria *Bacillus subtilis* from soil.

The microorganism *Bacillus subtilis* was selected for this study. *Bacillus subtilis* is obtained, isolated and grown from the soil at COMSATS University in Abbottabad, Pakistan. The soil sample was picked from a fresh vegetable dump area. A weight of 5 g of the soil sample was taken in 25 mL TBS, suspended, and diluted to the specific dilution factor. It was then plated on TSA and incubated for 24 h at 37 °C. The method that is represented in Figure 2 was used to isolate strains of true bacteria *Bacillus subtilis* from the soil by obtaining the grown colony and using it for identification. 

The technique used for the isolation of bacteria was the streak plate technique. It started with plating heat-treated soil suspensions on aldohexose mineral base agar in a cone-like flask with 10 g glucose, 1 g (NH_4_)_2_SO_4_ or KNO_3_, 0.8 g K_2_HPO_4_, 0.2 g KH_2_PO_4_, 0.5 g MgSO_4_ and 7H_2_O, 0.05 g CaSO_4_.7H_2_O, 0.01 g FeSO_4_.7H_2_O, and 1.2 g agar. Another was added to water to 1 L, and the pH scale was adjusted to seven. The square plate’s (petri dish) was incubated at 37 °C. White, round, sleek, and glossy colonies 1–3 mm in diameter developed on the nitrate (KNO_3_) medium in 24 h. However, not all strains use nitrate, so the advice is to use ammonium ion ((NH_4_)_2_SO_4_) medium in parallel. Rod-shaped colonies were detected by their look and the general giant cells of this species were confirmed microscopically. The details of the isolation of the bacteria from soil are shown in Figure 2.

**Figure 3:**
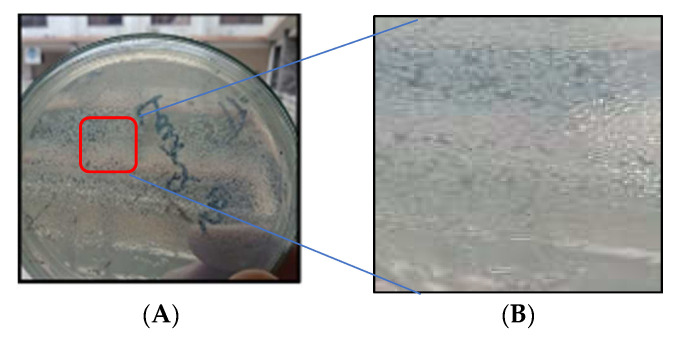
Growth of colonies. (**A**) Isolation sample from soil and (**B**) detailed image of colony.

Identification was performed using a polymerase chain reaction (PCR) test after extracting the deoxyribonucleic acid (DNA) and matching it. A morphological investigation was also conducted using a microscope for identification. After identification, the bacteria grew on a plate using the streak plate technique. For the streaking technique, first, the distilled water in the required volume, and nutrient agar and petri plates were placed for autoclaving (121 °C for 15 min), and then add nutrient agar (28 g/L) to the distilled water, and finally apply on the petri dish. After applying agar media on the plates in a laminar flow bath (UV light-treated for 10 min) the streaking petri plates were then placed in a static incubator (at 37 °C for 24 h) to verify the contamination. Afterward, the colonies of the bacteria were removed using a wire loop, first made red hot by using a spirit lamp in laminar flow, and streaked on the agar media plate. After the streaking, the plates were placed in a static incubator at (37 °C for 24 h) and checked for the growth of bacteria without contamination for further use in the process. The growth of the bacteria is shown in Figure 3.

After obtaining the true strain grew, a colony was chosen to be spread in a cone containing nutrient broth. The media containing peptone (0.5% *w*/*v*), glucose (0.5% *w*/*v*), and yeast extract (0.05% *w*/*v*) were incubated at 37 °C (120 rpm and 24 h) to rehydrate the bacterial strains in the nutrient broth media. After 24 h of growth of the bacteria in a shaking incubator, the colonies were counted using a UV-Vis spectrophotometer (OD600 nm). First, a blank sample of agar media was obtained (3 mL) in a glass cuvette and placed in the spectrophotometer (OD600 nm) set at zero for reading. Then the cuvette was filled with 3 mL of grown agar culture and placed in the spectrophotometer. A reading was taken and then put into Ramachandran’s equation for counting the bacterial colonies.

Y = 8.59 × 10^7^ X^1.3627^. (4)

**Figure 4:**
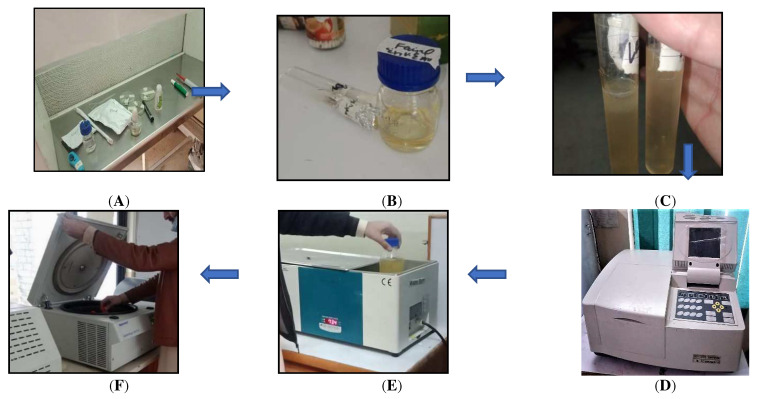
Process of obtaining bacterial spores. (**A**) Laminar flow preparation of nutrient broth; (**B**) bacterial growth medium nutrient broth; (**C**) shaking incubation growth; (**D**) spectrophotometer colony counter; (**E**) water Bath for growth inactivation; (**F**) centrifugation for obtaining bacterial spores.

After obtaining the required number of colonies, the mixture was placed in a water bath. To inactivate the harvested vegetative cells and obtain a pure suspension of spores, the mixture (media and bacteria) was placed in a water bath (80 °C for 10 min). The spore suspension was obtained by performing centrifugation for 15 min at a speed of 3000 rpm. Figure 4 shows the steps for obtaining spores of the bacteria. 

### 2.4. Iron Oxide Nanoparticle Immobilization Procedure

The obtained spores were then encapsulated in immobilization media to encase IONPs in concrete during casting. IONPs (250 µg/mL) were obtained and dispersed in distilled water using sonication for 15 min at 35 °C in a sonicator to create pores for the bacteria encapsulation. Then the mixture was transformed into a suspension containing bacterial cells for immobilization. For the bacteria to be encased in concrete during casting, food consisting of the nutrients calcium chloride anhydrous (40 g/L), urea (65 g/L), and yeast extract (2 g/L) was dissolved in distilled water. Then, the solution was inoculated with the immobilized bacteria IONPs, and the bacterial food was mixed and stirred for 2 min and gradually added to the concrete mixture.

### 2.5. Casting of the Concrete Specimens

The details of the bio-concrete cast in this study are listed in Table 1. In self-healing concrete, the ingredients are mixed with encapsulated bacteria along with precursor (urea, yeast extract, peptone and lab lemco powder) added into water. This suspension were added slowly into the concrete mix during mixing as a replacement for potable water. Microorganism incorporates in concentrations of 10^3^, 10^6^, and 10^9^ cells of true bacteria *Bacillus subtilis* per milliliter of water. Four concrete mixes—a control concrete and three concrete mixes, including the different concentrations of bacteria solution (10^3^, 10^6^, and 10^9^) cells/mL—were cast in molds of 150 mm × 150 mm × 150 mm.

The bacterium *Bacillus subtilis* is considered to be a harmless organism as it does not possess traits that can cause a disease or that are harmful to human beings [30]. Moreover, it is not considered pathogenic or toxigenic [31]. The inactive bacterial spores were encapsulated in nanoparticles and incorporated into the concrete mix. During the concrete hydration process, the bacteria remained inside the nanopellets, and therefore remained unaffected by the highly alkaline environment of the concrete. Furthermore, according to [31], this bacteria can survive at a pH of 14. These bacteria become active only when these nanopellets break [32].

### 2.6. Experimental Procedure

The normal consistency of cement was obtained to determine the amount of water to be added to the cement for a paste of normal solidity. Ordinary Portland cement (OPC) was used as a binder in all concrete types, and X-ray fluorescence (XRF) was used to obtain the chemical composition of OPC. The physical and chemical properties of the binder are mentioned in Table 2.

**Figure 5:**
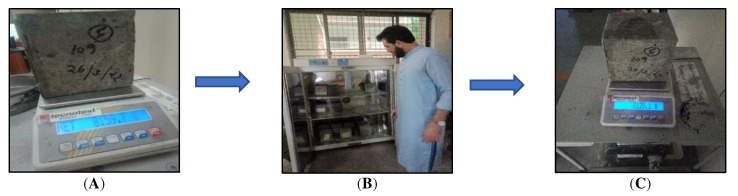
Water absorption technique. (**A**) Wet weight of the sample pre-cracking. (**B**) Pre-cracking samples drying in furnace. (**C**) Dry weight of the sample.

A slump test was performed to determine the consistency and workability of the cement concrete. A concrete is determined to be viable if it can be simply mixed, placed, compacted, and finished. A viable concrete should not show any segregation or damage. The control concrete and microorganism concrete mixtures were designed to have a slump of 25–75 mm. For the microorganism concrete mixture, cell concentrations of 10^3^, 10^6^, and 10^9^ cells of *Bacillus subtilis* per milliliter of water were used. Concrete cube specimens 150 mm × 150 mm × 150 mm in size were hardened to develop a 28-day compressive strength of 20 MPa. At 28 days, the specimens were tested for compressive strength using a compression testing machine. Avoiding any shocks, loads were applied at an increasing rate of approximately 140 kg/cm^2^/min until the specimen could not sustain the load. The maximum load was recorded, and the compressive strength was calculated by dividing the maximum load by the size of the cross-sectional area. This test was conducted on four specimens and the average value was taken. The test was conducted as per ASTMC 642-97 to work out the inflated resistance to water penetration in the concrete. The control and bacterial concretes were cast in cubic molds 150 mm in size. The square specimens were cured for 28 days. To determine the water absorption percentage for both the control and bacterial concrete, the specimens were placed in a water tank and cured for 28 days. For the bacterial concrete samples with varying concentrations of encapsulated IONP bacteria, both the pre-cracking and post-cracking wet weights were recorded using a scale, as shown in Figure 5. Then they were placed in an oven and dried at 35 ± 5 °C for 12 h. The water absorption of the specimens was calculated before drying and after drying for both the control specimens and bio-concrete samples with encapsulated iron oxide nanoparticles with varying concentrations of bacterial cells, i.e., 10^3^, 10^6^, and 10^9^ cells/mL of potable water.

**Figure 6:**
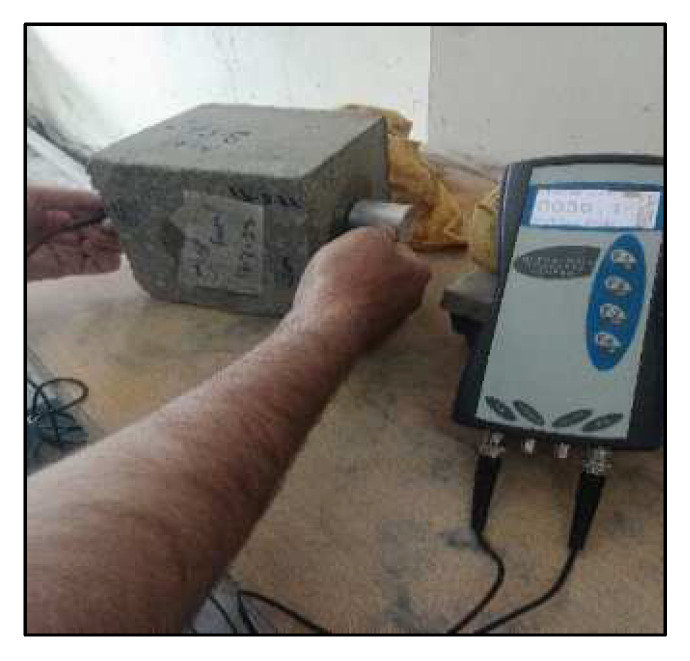
The UPV test of the concrete cube.

The ultra-sonic pulse velocity test as shown in Figure 6 was performed on both the control specimens and bio-concrete samples to detect the internal defects within the concrete matrix. 

### 2.7. Preparation of Cracks and Quantification of Crack Healing by Bacterium

The microorganism concrete cube specimen was cast with microorganism cell concentrations of 10^3^, 10^6^, and 10^9^ cells per milliliter of blending water with *Bacillus subtilis*. All the ingredients needed for the M20 concrete mixture were weighed and mixed. Further, the specimens were cast instantly with the admixture. Cracks were introduced until they were visible and measurable within the cube specimen by applying loads using a compression testing machine. The concrete was removed before the final setting of the concrete and a specified crack was clearly seen within the specimen. Then the specimen was placed for healing in a natural process tank and images were taken to ascertain the healing of the crack for a healing evaluation period of 28 days of immersed curing. The presence of white precipitates during healing evaluation period indicated that the crack was healing well.

### 2.8. Mix Proportions

The mix proportion ratios for the M20 ordinary concrete cubes were finalized as shown in Table 3. For the bacterial concrete, a superplasticizer was added as a 1% replacement for the cement, and bacterial spores were added as fine aggregate. Three mixtures of bacteria-based solutions were used for the bio-concrete preparation, and their mix designs are shown in Table 3.

## 3. Results and Discussion

### 3.1. Characterization of IONPs

**Figure 7:**
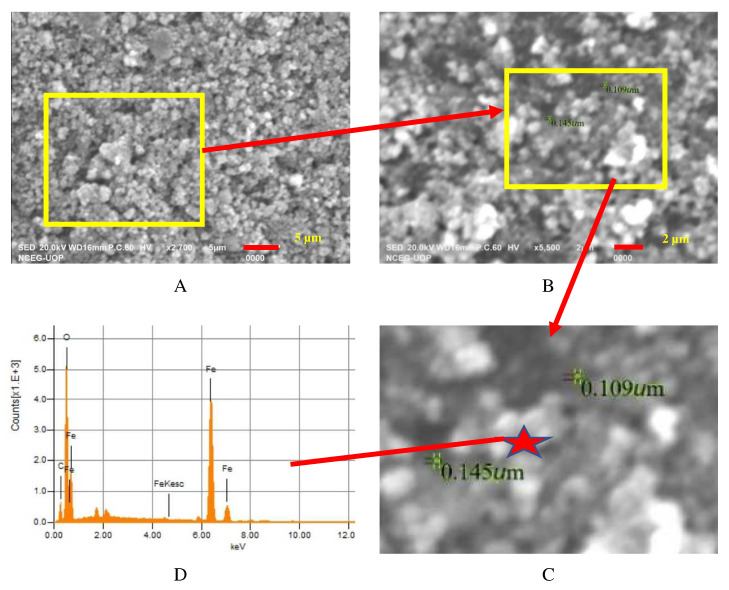
Analysis of synthesized IONPs in (**A**) SEM image zoom ×2700 and (**B**) SEM image zoom ×5500. (**C**) Size and location of EDX and (**D**) EDX analysis of synthesized IONPs.

The SEM analysis shows that the IONPs particles were small porous particles, rounded and spherical, as shown in Figure 7. In addition, the SEM images show that the synthesized IONPs were uniformly distributed and no agglomerates were seen. The porosity in the IONPs reflected in the morphological analysis is best suited for bacteria encapsulated in concrete. Magnetic nanoparticles with surface grafting may be readily diffused in water to generate a homogenous suspension. Similar SEM results can be found in the literature for synthesized IONPs [33]. The EDX spectra revealed significant Fe and O peaks. In the Fe_3_O_4_ compositional components generated through co-precipitation synthesis, the Fe had a value of 53.80%, while the O had a value of 32.30%. These data show that the synthesis products and samples were pure and had excellent stoichiometry. The SEM-EDX showing an FeO percentage of 89.55% (calculated quantitively from the microscale) reflect that the sample was pure, and no other impurity peaks were observed, as shown in Figure 7D. Similar results can be found in the literature [34].

**Figure 8:**
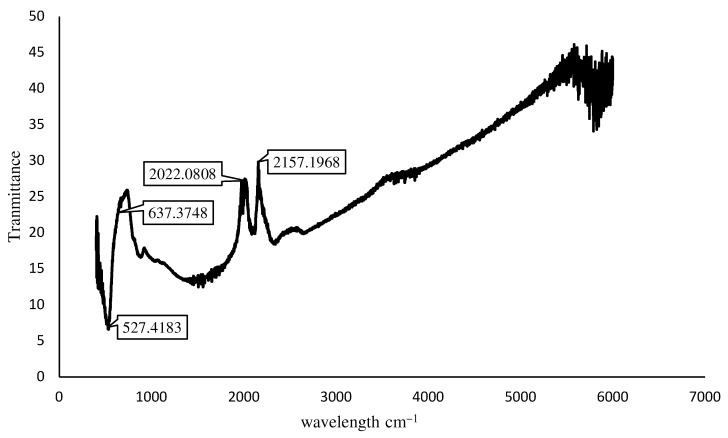
FTIR analysis of IONPs.

It can be seen in the FTIR spectra of the prepared Fe_3_O_4_ nanoparticles that the characteristic absorptions of the Fe-O bond are at 530 cm^−1^ and 634 cm^−1^. Both the peaks at 530 cm^−1^ and 628 cm^−1^ in Figure 8 confirm the existence of the Fe-O bond. The characteristic peak of 2157 cm^−1^ confirms the existence of carbon. The results in the literature [35] also confirm these results for Fe_3_O_4_ nanoparticles.

**Figure 9:**
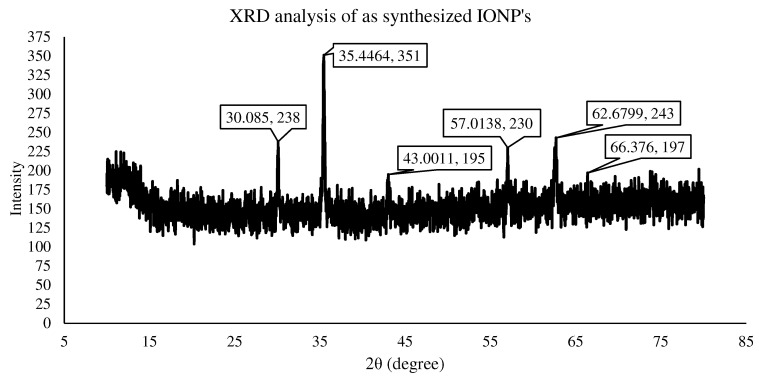
XRD analysis of synthesized IONPs.

The crystalline phases of the products were determined using XRD measurements. The Fe_3_O_4_ samples displayed six typical peaks at values of 30, 35.4, 43, 57, 62.67, and 66.37, which correspond to the (238), (351), (195), (230), (243), and (197) crystal planes, as can be seen in Figure 9. No more peaks occurred, suggesting the development of a clean and single phase devoid of impurities left over from the unreacted precursors of the production of other phases, such as Fe_3_O_4_ or Fe. Furthermore, the peaks are quite wide, indicating the creation of very tiny particles in the nano-scale region [36]. The average crystallite sizes (d), as determined by the Debye–Scherer equation d = Kλ/(cosθ), were around (a) 12.6 nm, (b) 13.4 nm, (c) 14.2 nm, and (d) 13.8 nm. The XRD pattern indicates that the Fe_3_O_4_ nanoparticles had a spinal structure with no distinctive impurity peak. Similar findings were discovered in the literature [34,35].

**Figure 10:**
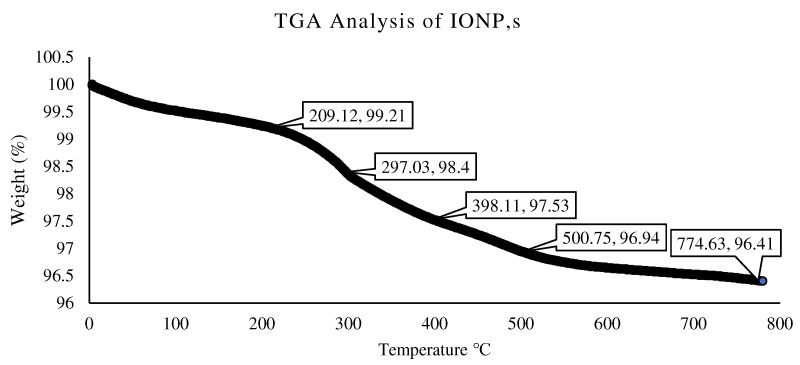
Shows the TGA analysis of IONPs.

The thermal stability of the IONPs was also determined. As IONPs were added to the concrete mix, the temperature within the concrete increased to 80–90 °C due to hydration reactions [37]. Therefore, it was deemed necessary to check the thermal stability of the IONPs. Moreover, IONPs are carriers of bacteria and they act as a shield for bacteria under high temperatures. The TGA graphs (Figure 10) show how the residual masses of the samples changed with the temperature. At high temperatures, the organic components and magnetite in the samples were totally burnt, creating gas products and transforming them into Fe_3_O_4_. The first weight loss of less than 1% up to 200 °C can be attributable to the existence of residual physisorbed water-based solution solvents. A weight loss of roughly 2% was observed for nanoparticles generated by chemical precipitation at temperatures ranging from 300 °C to around 800 °C. This indicates that unreacted sodium acetate existed within the nanoparticles.

### 3.2. Compressive Strength

**Figure 11:**
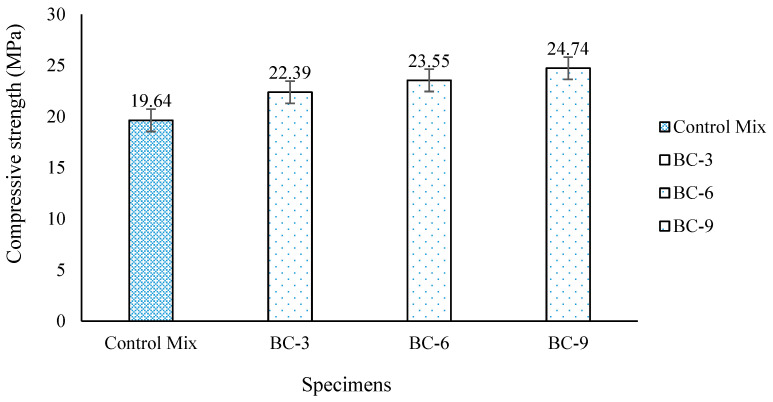
Compressive strength analysis of bio-concrete and control mix.

The compressive strength was determined at 28 days after casting the control M20 concrete mix. After 28 days of immersed curing, the average result of the compressive strength was recorded at 19.64 MPa. The average compressive strength of the control mix was considered a reference for comparison with the compressive strength results of the bio-concrete. The compressive strength results of the bio-concrete show an increase in the strength gain compared to the control concrete mix, as shown in Figure 11. The bio-concrete with 10^9^ cells/mL (BC-9) shows more gain in the compressive strength compared to the bio-concretes with 10^6^ (BC-6) and 10^3^ (BC-3) cells/mL, respectively, as shown in Table 4. The bacterial precipitation of calcium carbonate is affected by the concentration of bacteria in a bio-concrete. The results confirm that increases in the concentration of bacteria cells in the concrete resulted in greater precipitation of the microbially induced calcium carbonate. The results show a higher strength gain in BC-9 by about 25.9% compared to the control mix due to the higher precipitation of calcium carbonate. The optimum gain in compressive strength was found to be 25.9% in BC-9 compared to BC-6 with 19.9%, and 14% in BC-3. The results show that bacterial load increments up to a specific limit can enhance the crack healing of bio-concrete. According to the experimental findings, concrete with a bacterial concentration of 10^9^ cells/mL healed wider cracks than concrete with a bacterial concentration of 10^6^ cells/mL. In addition, the results demonstrate that with increases in the bacterial concentration, the calcium carbonate precipitation also increased.

Bacteria concentrations up to 10^6^ cells/mL had no impact on the quality of concrete, according to the UPV test results (Table 3). The quality of the concrete decreased, according to the results with 10^9^ cells/mL of bacterial concentration solution. This could increase the permeability of the bio-concrete. Overall, the concrete UPV test results are satisfactory; all of the concrete mixes were in excellent to very good condition, with only a few exceptions in good condition. With increases in the porosity of the bio-concrete, the bacteria may grow faster and efficiently, resulting in increased precipitation of microbially induced calcium carbonate. The fact is that in the pores, the entry of oxygenated water provides support for the bacterial precipitation of microbial calcium carbonate. The results show (Table 4) that the average water absorption decreased with increases in the concentration of the bacterial load in the bio-concrete. With increases in the bacterial load, more microbially calcium carbonate resulted in blocking the pores.

### 3.3. Self-Healing Capability of Bacteria Bacillus Subtilis Immobilized with IONPs

**Figure 12:**
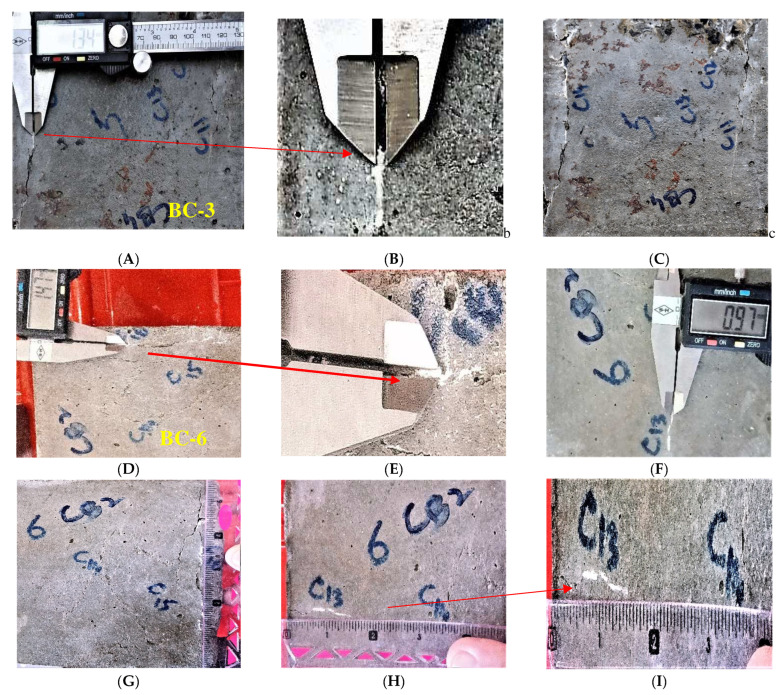

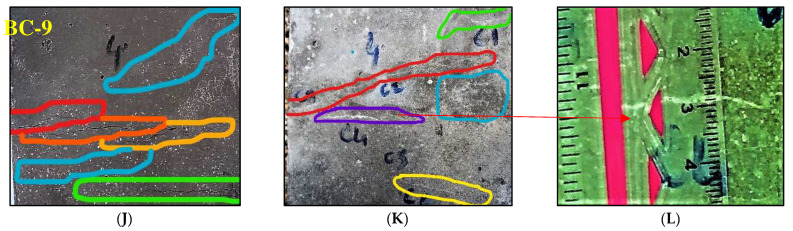
Crack healing of bio-concrete; (BC-3) (**A**) crack healing of 1.34 mm; (**B**) digitized image of healed crack; (**C**) crack healing of bio-concrete; (**D**) (BC-6) crack healing of 1.47 mm; (**E**) digitized image of healed crack; (**F**) crack healing of 0.97 mm; (**G**) crack healed length (C-16) 5.3″; (**H**) crack healed length (C-13) 3.5″ (**I**) digitized image of healed crack; (**J**) cracking of BC-9; (**K**) crack healed; (**L**) crack healed width (C-4) 1.71 mm.

The healing of created cracks after applying loads on the concrete specimens with bacterial concentrations of 10^3^, 10^6^, and 10^9^ cells/mL are shown in the Figure 12. The concentration of bacteria in the bio-concrete influenced the bacterial precipitation. The results confirm (Figure 12) that increases in the concentration of bacteria cells in the concrete resulted in the increased precipitation of microbially induced calcium carbonate. The wider crack of 1.71 mm was entirely repaired by BC-9, followed by BC-6 with a 1.47 mm crack, and BC-3 with 1.34 mm. Similar patterns were seen in the crack length, with BC-9 recovering 5″ (127 mm), BC-6 recovering up to 3.5″ (89 mm), and BC-3 recovering up to 3″. (76 mm). The crack width influenced the crack length healing. The crack length recovery was affected by the crack width. Different ranges of crack length were restored entirely when the crack width was up to 0.60 mm. All specimens with various bacterial concentrations (BC-3, BC-6, and BC-9) healed cracks in concrete to a maximum length of 5.6 inches and a crack width of 0.60 mm. When the crack width increased, the crack length recovery of BC-3 decreased, followed by BC-6. A crack width up to 1.71 mm was completely recovered by BC-9. At a crack width of 1.47 mm, the crack length was partially healed by BC-6, as shown in the Figure 12. Similarly, for a crack width of 1.34 mm, the crack length was partially healed by BC-3.

### 3.4. Water Absorption

**Figure 13:**
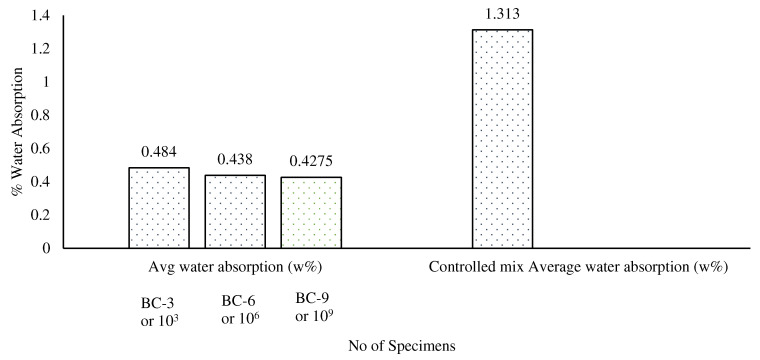
Comparative analysis of water absorption of control mix and bio-concrete mix.

The results show (Figure 13) that the average water absorption decreased with increases in the concentration of the bacterial load in the bio-concrete. Overall, BC-9 showed 68% less water absorption compared to the control specimen. This is due to the fact that an increase in the bacterial load caused more calcium carbonate to be produced by the microbes, which blocked the pores and increased the concrete’s durability.

### 3.5. Characterization of Microbially Induced CaCO_3_

**Figure 14:**
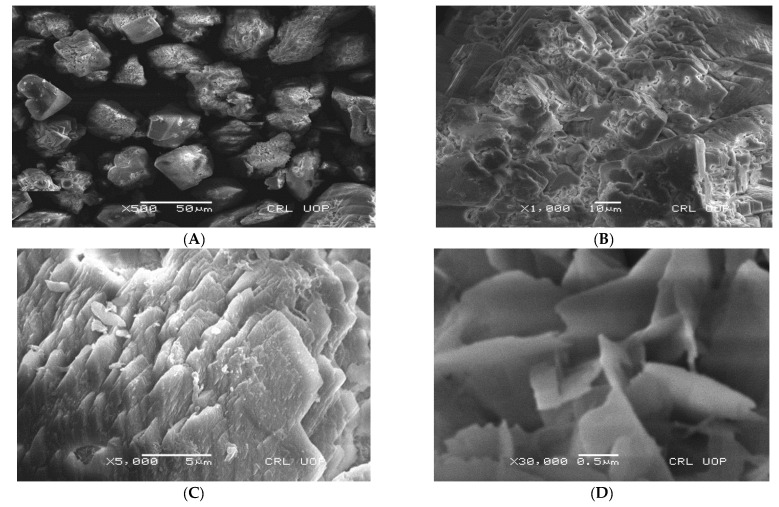
SEM analysis of microbially induced CaCO_3_ at different resolutions (**A**) ×500; (**B**) ×1000; (**C**) ×5000; (**D**) ×30,000.

The SEM analysis of the microbially induced CaCO_3_ shows that the particles were a cubical shape, which confirms the calcite morphology (Figure 14). The calcite was in the stable form of calcium carbonate compared to the other allotropic form of calcium carbonate. The stability of the allotropic form is a good sign of the durability of the concrete. Due to the stronger bonding of the precipitated calcite, the allotropic form of calcium carbonate, it had higher water resistance and was not soluble in water. In addition, the calcite is thermally stable until 500 °C, as seen in the literature [38]. The findings also revealed that the precipitated calcite had fewer pores, indicating a tight bond between it and the other concrete constituents during the crack repair. Reduced permeability is the result of higher density and fewer pores. A decrease in the permeability of the bio-concrete due to calcite precipitation increased the durability. In addition, it had higher density and higher compressive strength results.

**Figure 15:**
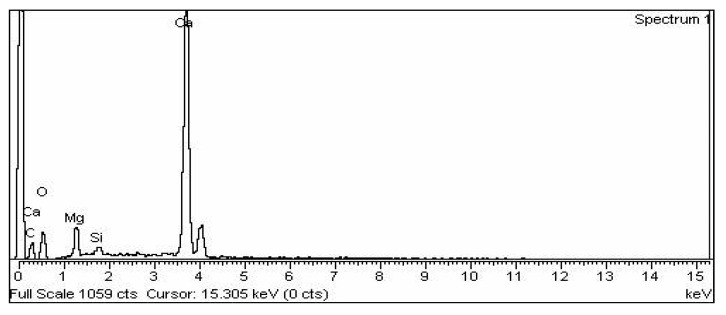
EDX analysis of microbially induced calcium carbonate (MICP).

The EDX examination of the microbially created CaCO_3_ confirms that the principal constituents found in the created powder were calcium, oxygen, and carbon atoms. The constituent compositions of the bio-precipitates closely matched those of pure CaCO_3_ crystals, indicating that the particles were produced by immobilized bacteria with IONPs. A similar finding can be seen in the literature [29]. Figure 15 shows the EDX of microbially induced calcium carbonate.

**Figure 16:**
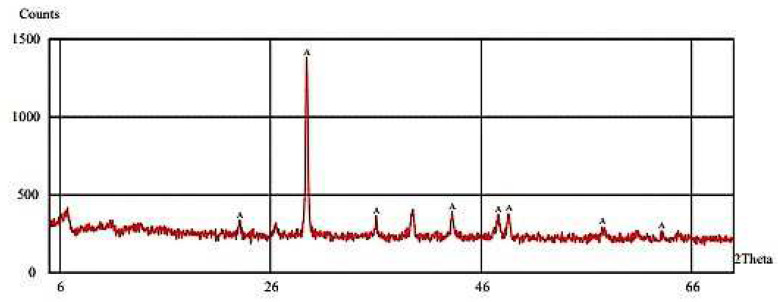
XRD analysis of microbially induced calcium carbonate.

The XRD analysis of microbially induced CaCO_3_ shows nine characteristic peaks labeled A at angles of 21, 29, 32, 36, 41, 48, 49, 57, and 61, as shown in Figure 16. The white precipitate was confirmed to be CaCO_3_ by XRD examination, with calcite (2θ = 29.3°) being the most common polymorph formed in the bio-concrete samples. A similar outcome can be found in the literature [29].

**Figure 17:**
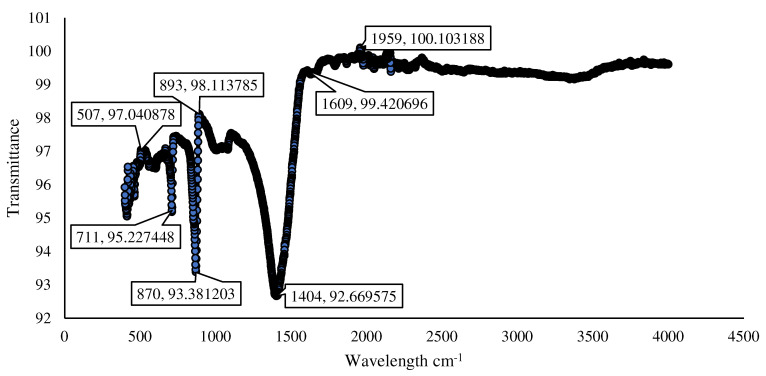
FTIR analysis of microbially induced calcium carbonate.

The FTIR analysis of microbially produced calcium carbonate in powder appears to be a useful approach for early identification, as shown in Figure 17. Calcite was the most abundant mineral generated by *Bacillus subtilis* cells (as determined by the intensity values of the bands at 873 cm^−1^ and 1800 to 1550 cm^−1^). The white precipitate deposited by *Bacillus subtilis* produced extremely distinctive acute stretching bands of calcites from 1800 to 1550 cm^−1^ [39,40]. 

## 4. Conclusions

It was established that bacterial immobilization using IONPs was a viable technique for addressing cell vulnerability in the construction of self-healing bio-concrete. The following conclusions can be drawn from this investigation.

The compressive strength of the samples improved with the addition of *B. subtilis* bacteria to the cement concrete mix, which changed with the concentration of bacteria employed in the cementitious material. The bio-concrete with 10^9^ cells/mL (BC-9) shows a 25.9% increase in compressive strength. The *B. subtilis* microorganisms have a great potential to produce CaCO_3_ for healing nano-/micro-scale structural/nonstructural cracks in cementitious composites, as evidenced by the repair of micro-cracks to wider cracks of 1.71 mm and a crack length of 127.0 mm completely. The creation of a protective layer surrounding bacterial spores is responsible for the contradictory behavior of IONPs that contributes to successful healing at older ages. The mechanical method was used in this work to quantify the self-healing of cracks. Additional research employing stress–strain data, as well as fracture origin and development pattern, may be performed in order to better understand the self-healing mechanism. The researchers also recommend expanding the investigation in the future to include complex bio-based analyses to determine the concentrations of consumed and conserved microorganisms at different times.

## Figures and Tables

**Table 1:** Bio-concrete cast in this study.

Encapsulation Material	Application	Precursor	Temperature (°C)	Type of Bacteria
Iron oxide nanoparticles	As a crack healer	Urea	Room temperature (27 °C)	*Bacillus subtilis*

**Table 2:** Chemical and physical properties of cement.

Chemical Composition	Content (%)	Physical Properties	Contents (%)
CaO	65.81	Insoluble residue (% mass)	0.55
SiO_2_	18.83	Specific gravity (g/cm^3^)	3.15
Al_2_O_3_	6.94	Specific surface area (m^2^/g)	0.83
Fe_2_O_3_	3.47	Particle size (d50) (µm)	16.58
MgO	1.94	Loss on ignition (% mass)	2.21
SO_3_	1.32		
Na_2_O + K_2_O	1.20		

**Table 3:** Mix proportions of control mix and bio-concrete.

Sample	Fine Aggregate(kg/m^3^)	Coarse Aggregate(kg/m^3^)	*Bacillus subtilis* (Cells/mL)	Urea (g/L)	Yeast Extract (g/L)	Calcium Chloride Anhydrous (g/L)	IONPs (µg/L)	Portland Cement (kg/m^3^)	Super-Plasticizer (kg/m^3^)	*w*/*c* Ratio
Control mix	945.87	1316.6	-	-	-	-	-	574.2	5.80	0.42
BC-3	945.87	1316.6	2.8 × 10^3^	65	2	40	250	574.2	5.80	0.42
BC-6	945.87	1316.6	2.8 × 10^6^	65	2	40	250	574.2	5.80	0.42
BC-9	945.87	1316.6	2.8 × 10^9^	65	2	40	250	574.2	5.80	0.42

**Table 4:** Strength analysis of bio-concrete before and after cracking.

Sample	80% Applied Load (Mpa) after 28 Days of Curing	Crack Healing Strength (Mpa) After 28 Days	UPV		% (w) Absorption (gm)	Density (kg/m^3^)
			Value	Remarks		
Control mix	19.45	-	4.43	v. good	1.345	2428.53
	23.76		4.51	excellent	1.057	2383.34
	15.72		4.13	v. good	1.36	2487.76
BC-3	10.64	14.68	4.12	v. good	0.412	2423.43
	16.37	27.70	4.39	v. good	0.556	2520.20
	21.03	24.79	4.41	v. good	0.434	2408.16
BC-6	13.99	29.06	4.46	v. good	0.352	2532.62
	9.35	14.61	3.98	good	0.524	2496.53
	23.92	26.98	4.15	v. good	0.374	2438.7
BC-9	11.75	23.09	4.29	v. good	0.468	2425.21
	8.45	17.58	3.68	good	0.387	2408.32
	20.6	33.56	4.13	v. good	0.362	2413.14

## Data Availability

Not applicable.
